# PLAU and LAMC2 can predict a poor prognosis in patients with HNSCC

**DOI:** 10.7150/jca.84407

**Published:** 2023-06-04

**Authors:** Zhi-chen Guo, Si-li Jing, Hao Cui, Lin-yang Xie, Si-jia Na, Jun-bo Tu

**Affiliations:** 1 Key Laboratory of Shanxi Province for Craniofacial Precision Medicine Research, College of Stomatology, Xi'an Jiaotong University, Xi'an 710004, China.; 2 Laboratory Center of Stomatology, College of Stomatology, Xi'an Jiaotong University, Xi'an 710004, China.; 3 Department of Oral and Maxillofacial Surgery, College of Stomatology, Xi'an Jiaotong University, Xi'an 710004, China.; 4 Department of Ophthalmology, The First Affiliated Hospital of Xinjiang Medical University, Urumqi, 830001, China.

## Abstract

**Objectives:** Head and neck squamous cell carcinoma (HNSCC) is the most common malignancy of the head and neck. However, the molecular mechanisms governing the development of HNSCC have not been fully elucidated.

**Materials and Methods:** Differentially expressed genes (DEGs) were screened out from The Cancer Genome Atlas (TCGA) and GSE23036 datasets. Weighted gene coexpression network analysis (WGCNA) was used to reveal the correlations among genes and to search for significantly correlated gene modules. The expression levels of genes in HNSCC and normal samples according to antibody-based detected methods was assessed by utilizing the Human Protein Atlas (HPA). The impact of the selected hub genes on the prognosis of HNSCC patients was assessed by analysing immunohistochemistry (IHC) and immunofluorescence (IF) expression levels and clinical data.

**Results:** Twenty-four genes positively correlated with tumour status and 15 genes negatively correlated with tumour status were screened out by WGCNA. PLAU and LAMC2 were associated with a poor prognosis in patients with HNSCC and were finally screened out and verified by GEPIA and HPA database analysis. Immunohistochemistry of samples collected from 175 patients with HNSCC and subsequent statistical analysis also showed that PLAU and LAMC2 were associated with a poor prognosis in patients with HNSCC, and the levels of these two factors were positively correlated. The expression and co-localization of PLAU and LAMC2 in HNSCC tissues were confirmed by double immunofluorescence labeling.

**Conclusions:** There was a positive correlation between PLAU and LAMC2 expression in HNSCC samples, and PLAU and LAMC2 might be independent prognostic biomarkers for HNSCC.

## Introduction

Head and neck squamous cell carcinoma (HNSCC) is the most common malignancy of the head and neck, accounting for more than 800,000 new cases and 450,000 deaths in 2018 worldwide 1. In the United States, 54,000 new HNSCC cases were diagnosed, and 11,230 patients died from HNSCC in 2021 2. HNSCC has gradually become a serious problem worldwide, and despite the rapid development of clinical treatment in the past few decades, the 5-year survival rate of this disease has not significantly improved 3. Given the high incidence and mortality, early detection and risk assessment can improve the prognosis of HNSCC. Therefore, better prognostic biomarkers are needed to identify early-stage disease, and key gene-targeted therapy is necessary and urgent to improve the survival rate of HNSCC patients.

The Gene Expression Omnibus (GEO) and The Cancer Genome Atlas (TCGA) databases have recently been extensively used to identify key differentially expressed genes (DEGs) associated with the prognosis of HNSCC 4-6. However, most studies only focused on a single database and screened out DEGs but ignored the combined analysis of multiple databases. In addition, the complicated network of the genome and the high degree of interconnection between function‐related genes were ignored. Currently, systematic bioinformatics methods are widely used in the research of various kinds of cancers 7-9.

In this study, we first aimed to combine the GEO and TCGA databases for analysis and then screen out the key DEGs of HNSCC in these two databases. Second, we constructed a coexpression network of related genes and a protein-protein interaction (PPI) network based on the DEGs of HNSCC and identified key genes deserving further investigation for their potential as biomarkers. An integrated bioinformatics analysis was used to further investigate the functions, pathways, and regulation mechanisms and ultimately screened out the key DEGs related to HNSCC prognosis. In addition, we verified the key DEGs by immunohistochemistry (IHC) combined with clinical data, and their effect on the prognosis of HNSCC patients was analysed.

## Materials and methods

### Patient selection

One hundred seventy-five formalin-fixed, paraffin-embedded blocks of HNSCC samples and 10 normal samples were collected from the Department of Oral and Maxillofacial Surgery, College of Stomatology, Xi'an Jiaotong University, between March 2017 and March 2022. The screening criteria were as follows: (1) patients were pathologically diagnosed with HNSCC; (2) patients had undergone surgical resection; (3) patients had complete clinical data and follow-up data; (4) patients did not have other malignant tumours. The use of HNSCC samples was approved by the ethics review board (approval no. xjkqll[2022]NO.028). The TNM and clinicopathological classification and staging of patients with HNSCC were performed according to the American Joint Committee on Cancer (AJCC) guidelines 10. Clinical data, including age, gender, survival status, differentiation, TNM stage, clinical stage, and recurrence were collected.

### Data collection and preprocessing

**Figure 1:**
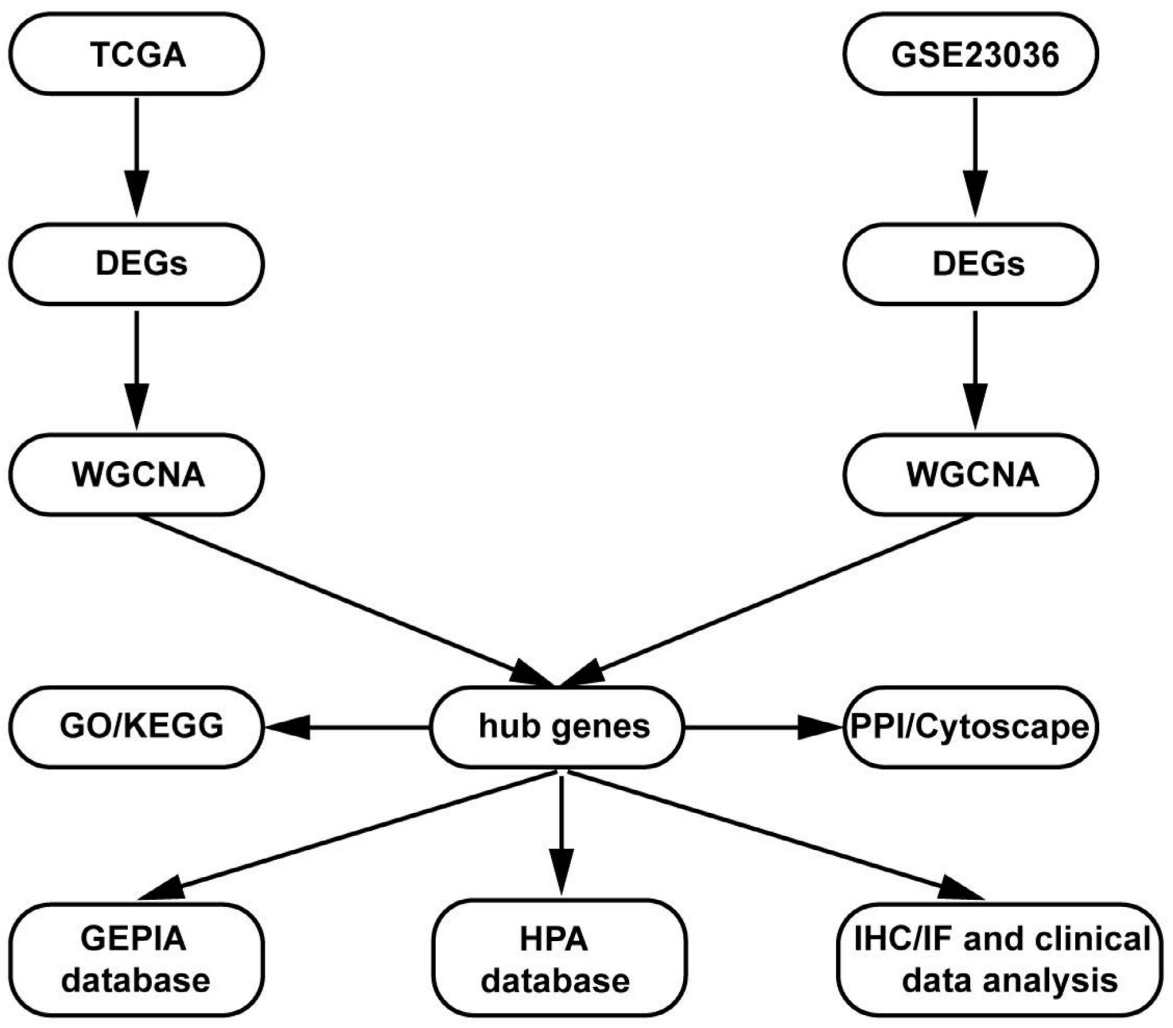
Flow diagram of the dates of processing and analysis.

The present study design is shown in the form of a flow diagram in Figure 1. The version of TCGA used in this study was updated in April 2022 (https://portal. gdc.cancer.gov). The TCGA is a comprehensive and coordinated project designed to improve diagnostic methods and treatment standards and ultimately prevent cancer. Information about sequencing and the pathological data of more than 30 kinds of human tumours can be analysed using TCGA 11. The gene expression data and corresponding clinical data of HNSCC samples were downloaded from the TCGA data portal. The RNA sequencing (RNA-Seq) FPKM data of 270 HNSCC samples and 19 adjacent normal samples were downloaded. The location of squamous cell carcinoma (1. lip; 2. palate; 3. gum; 4. base of the tongue; 5. other and unspecified parts of the mouth; 6. floor of the mouth; 7. other and unspecified parts of the tongue; 8. oropharynx) were obtained from the TCGA database for further analysis. Then, differential expression analysis was performed in the R software “edgeR” package with the cut-off criteria of |log2 (fold change [FC])| > 1.0 and false discovery rate (FDR) adjusted *P* value < 0.01.

GEO (http://www.ncbi.nlm.nih.gov/geo) is a public functional genomics data repository containing array and sequence-based data. The GSE23036 12 dataset (Affymetrix HG-U133A 2.0 Array; Affymetrix, Santa Clara, CA), with a total of 63 HNSCC samples and 5 normal mucosa samples, was downloaded from GEO to screen the DEGs. The probe ID was converted into an international standard name for gene symbols using Perl programming.

### DEGs screening in both the TCGA and GEO databases

We used the “limma” and “edgeR” R packages to screen out the DEGs between HNSCC samples and normal samples from the TCGA database 13 and the GEO database 14. Adjusted *p* value < 0.05 and |log2FC| >1) were chosen as the cut‐off thresholds.

### Coexpression analysis of DEGs in HNSCC in both databases

Weighted gene coexpression network analysis (WGCNA) is an algorithm that is based on high‐throughput gene expression profiles and is widely used in gene coexpression network identification in various diseases to reveal the correlation of genes and to search for significantly correlated gene modules. In this study, the coexpression analyses were conducted using the “WGCNA” R package 15 with TCGA and GEO data and patients' corresponding clinical data. The power of β = 15 (scale-free R2 = 0.83) and a cut‐off module size = 30 were set as the soft threshold to ensure a scale-free network. Gene significance and module membership were statistically calculated in an intramodular analysis of the module, and the genes in the hub module with a correlation ≥ 0.8 were selected as hub genes. Significant modules were defined with a *p* value < 0.05.

### Functional annotation and pathway analysis

Gene Ontology (GO) and Kyoto Encyclopedia of Genes and Genomes (KEGG) enrichment analyses were performed using the “colorspace” and “string” R packages and the Bioconductor packages “DOSE”, “clusterProfiler”, and “Pathview” 16. Each module in WGCNA was analysed in Search Tool for the Retrieval of Interacting Genes (STRING) version 11 (https://string-db.org/) and Cytoscape software. The STRING online database and a combined score > 0.7 were used for PPI network construction. The top 10 hub genes were visualized using Cytoscape software.

### Validation and analysis of the hub genes

The significant intersecting genes from the hub modules in WGCNA were identified as hub genes. Further validation and survival analysis of these hub genes were performed by using the Gene Expression Profiling Interactive Analysis (GEPIA) database (http://gepia2.cancer-pku.cn/index.html) 17. We plotted the survival curves and expression levels of the hub genes in the GEPIA database. The Human Protein Atlas (HPA) (https://www.proteinatlas.org) is a website that contains IHC-based expression data for nearly 20 highly common kinds of cancers, and each tumour type includes 12 individual tumours 18. In this study, a direct comparison of the protein expression of the selected hub genes between normal oral mucosa and HNSCC samples was performed by IHC imaging.

### Immunohistochemical and immunofluorescence staining

HNSCC samples and normal samples were collected from humans for IHC; the methods for IHC are described in our previous study 19. IHC was performed with anti-PLAU (17968-1-AP, Proteintech, 1:500) and anti-LAMC2 (ab210959, Abcam, 1:500) antibodies. The methods of immunofluorescence staining of PLAU and LAMC2 were similar to those of IHC. Sections were subjected to rehydration and antigen retrieval, followed by elimination of autofluorescence. Primary antibodies were then applied and incubated at 4°C overnight, followed by fluorescence-conjugated secondary antibody incubation and DAPI staining of nuclei.

### Evaluation of ICH staining

Image-Pro Plus version 6.0 software (Media Cybernetics, Inc., Bethesda, MD, USA) was used to evaluate the intensity score (IS) of IHC by calculating the integrated optical density (IOD) three times in each field, and the IOD/I of the total area of each field was also calculated simultaneously. According to the IOD value, the staining IS values were 0 (-), 1 (+), 2 (++), and 3 (+ + +). We divided the staining proportion score (PS) into four levels: 0 (0%), 1 (1-25%), 2 (26-50%), 3 (51-75%); and 4 (76-100%). The IS multiplied by the PS was the final result of the staining score 20. The expression of PLAU and LAMC2 was categorized into a low expression group (0-6) and a high expression group (7-12) in this study according to the staining score results.

### Statistical analysis

SPSS 25.0 software (IBM) was used for statistical analysis. For analysis of clinical data, counting data were expressed by frequency and composition ratio, the chi-square test was used for comparison between groups, and Cox regression was used to analyse the correlation between clinical indicators and death. Statistical significance was defined as *P* < 0.05 (*P < 0.05, **P < 0.01, and ***P < 0.001).

## Results

### Identification of DEGs

**Figure 2:**
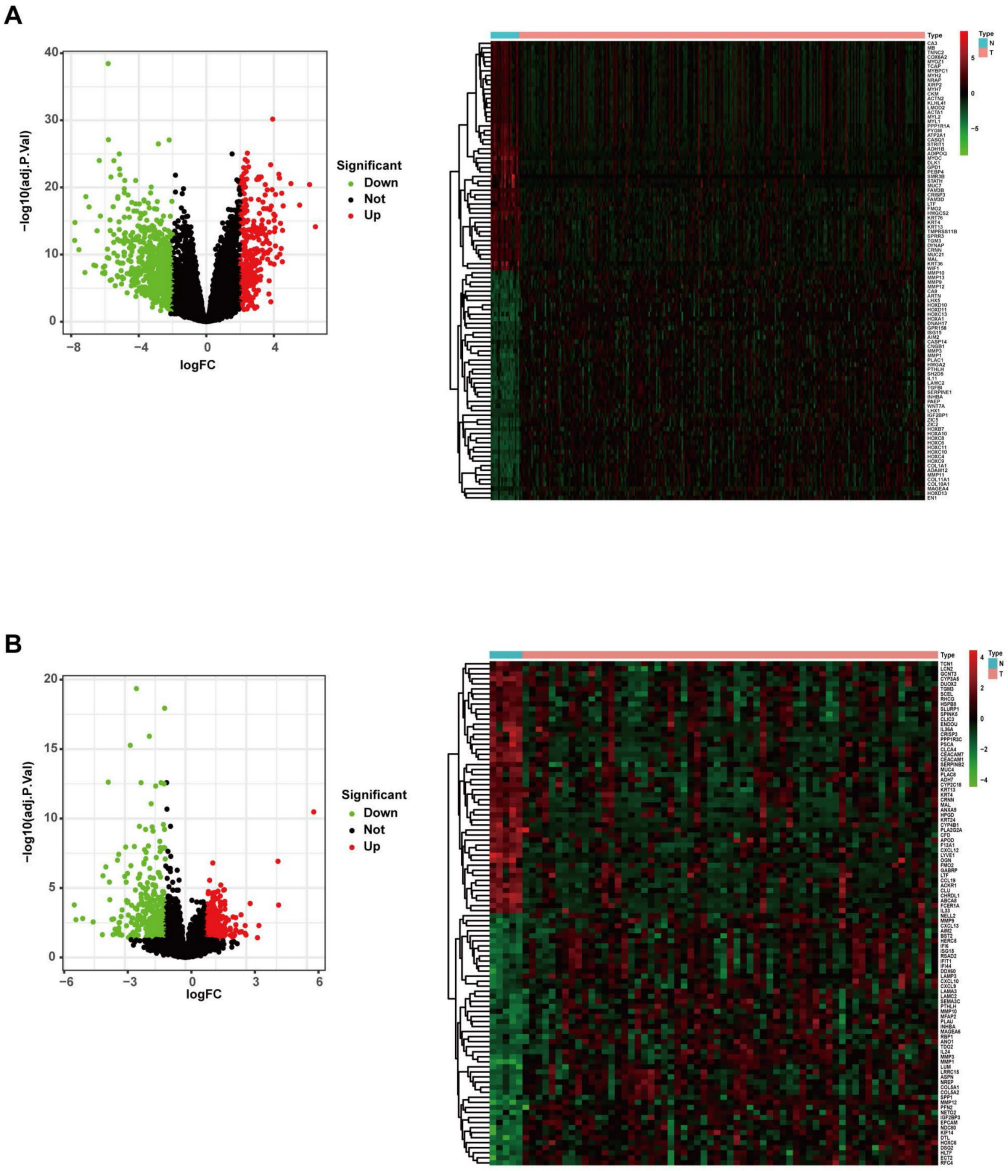
Differentially expressed genes in the TCGA and GEO datasets. (A) Volcano map and heatmap of significantly differentially expressed genes between HNSCC and normal samples in the TCGA dataset. (B). Volcano map and heatmap of significantly differentially expressed genes between HNSCC and normal samples in the GEO dataset.

The gene expression profiles of the TCGA RNA-Seq FPKM data of 270 HNSCC samples and 19 adjacent normal samples were analysed using the “limma” package of R software. A total of 952 DEGs were identified (293 upregulated and 659 downregulated), and the volcano plot of all DEGs and the heatmap of the top 50 DEGs are shown in Figure 2A. The gene expression profiles of the GSE23036 dataset from the GEO database were also analysed using the “limma” package of R software. A total of 570 DEGs were identified (236 upregulated and 334 downregulated). The volcano plot of all DEGs and the heatmap of the top 50 DEGs are shown in Figure 2B.

### Construction of a weighted gene coexpression network and identification of key modules

**Figure 3:**
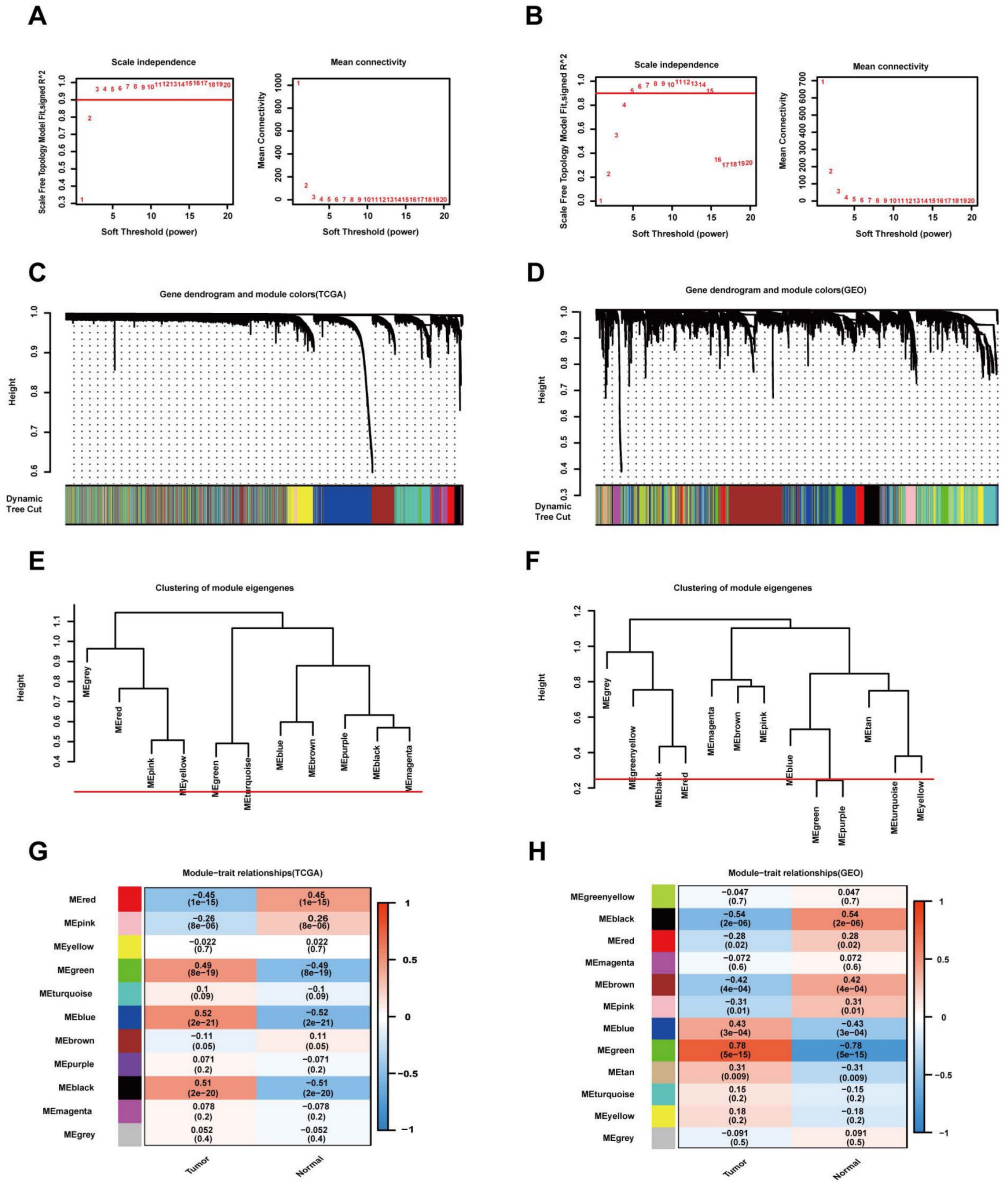
WGCNA modular analysis of the TCGA and GEO datasets. (A, B) Analysis of the two datasets of the scale-free fit index for various soft-thresholding powers (β) and the mean connectivity for the soft-thresholding powers. (C, D) DEG clustering and module screening based on gene expression patterns in the two datasets. (E, F) Clustering of module eigengenes. A cut-line (0.25) was selected for the module dendrogram, and some modules were merged according to the dissimilarity of estimated module eigengenes. (G, H) Correlation module heatmap of tumour samples compared to normal samples. The blue gene module was most positively associated with tumour samples, and the red module was negatively associated with tumour samples in the TCGA dataset. The red gene module was most positively associated with tumour samples, and the black module was negatively associated with tumour samples in the GEO dataset.

The "WGCNA" package in R was used to place the DEGs with highly relevant expression patterns into modules by average linkage clustering. A soft-thresholding procedure was performed by WGCNA, and a best-fit cut-off value (β = 13) was selected at the lowest mean connective value and appropriate scale-free topology fit index (0.75) (Figure 3A, B). A total of 11 modules were identified in the TCGA database (Figure 3C), and 12 modules were identified in the GEO database (Figure 3D). We set the MED threshold as 0.25 to merge similar modules (Figure 3E, F). In the TCGA and GEO databases, the module-trait heatmap revealed the differences in the expression of different module gene sets between tumour and normal samples. In the TCGA database, the blue module was positively significantly correlated with tumour status (R = 0.52 and *P* = 2e-21), while the red module was negatively significantly correlated with tumour status (R = -0.45 and *P* = 1e-15) (Figure 3G).

**Figure 4:**
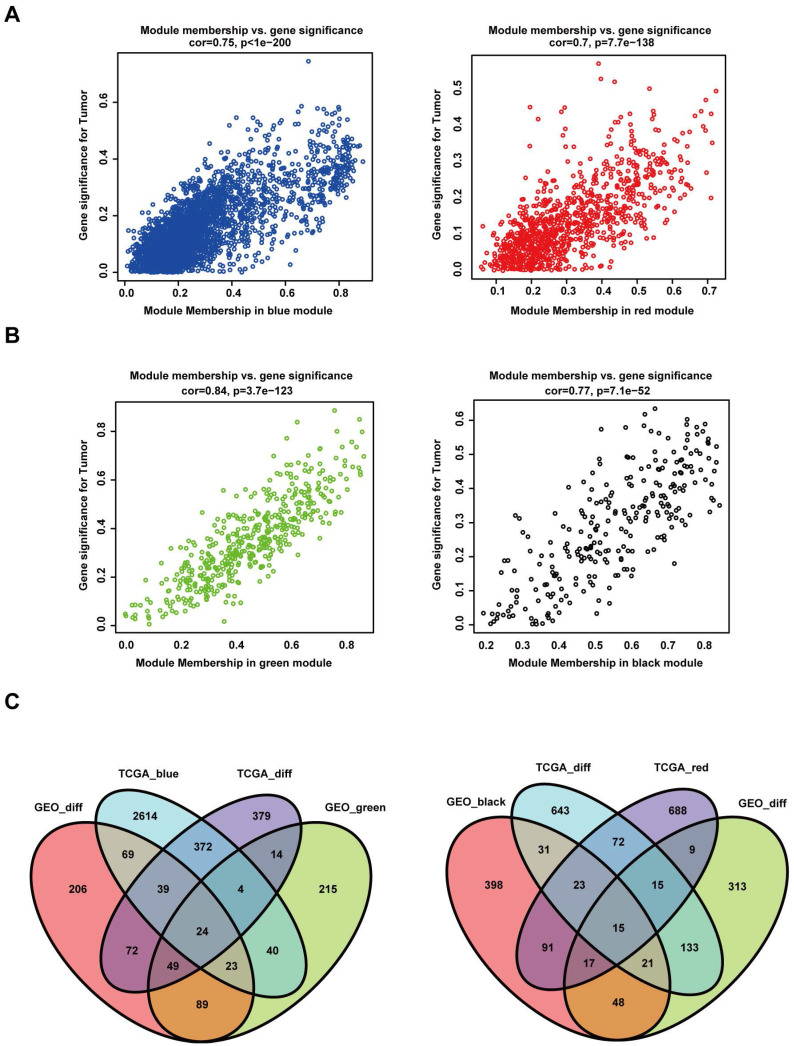
Intersecting DEGs of different modules in both the TCGA and GEO datasets. (A) Relationship between module membership in the blue and red modules and gene significance for the tumour in the TCGA dataset. (B) Relationship between module membership in the green and black modules and gene significance for the tumour in the GEO dataset. (C) In the left panel, the gene sets were screened out by intersecting the genes positively associated with the tumour and the DEGs. In the right panel, the gene sets were screened out by intersecting the genes negatively associated with the tumour and the DEGs.

In the GEO database, the green module was positively significantly correlated with tumour status (R = 0.78 and *P* = 5e-15), while the black module was negatively significantly correlated with tumour status (R = -0.54 and *P* = 2e-06) (Figure 3H). In the TCGA database, the intramodular analysis showed that genes in the blue module were highly positively correlated with tumour status (cor = 0.75, *P* < 1e‐200), and those in the red module were highly negatively correlated with tumour status (cor = 0.7, *P* = 7.7e‐138) (Figure 4A). In the GEO database, the intramodular analysis showed that genes in the green module were highly positively correlated with tumour status (cor = 0.84, *P* = 3.7e‐123), and those in the black module were highly negatively correlated with tumour status (cor = 0.77, *P* = 7.1e‐52) (Figure 4B).

To identify the common DEGs that were positively correlated with HNSCC in the TCGA and GEO databases, we intersected a total of 24 genes from four gene sets. In addition, for the common DEGs negatively correlated with HNSCC in the TCGA and GEO databases, we intersected a total of 15 genes from four gene sets (Figure 4C).

### GO and KEGG analyses

The gene sets of 24 positively and 15 negatively correlated DEGs were subjected to GO and KEGG pathway analyses to further explore their biological functions. For the 24 positively correlated DEGs of the gene sets, a total of 30 GO terms and 9 pathways (*P* < 0.05) were identified. The most significantly enriched biological process, cellular component, and molecular function of the GO terms were the regulation of the extrinsic apoptotic signalling pathway in the absence of ligand, the laminin complex, and the extracellular matrix structural constituent, respectively. Similarly, the significant pathways were mainly enriched in the ECM-receptor interaction signalling pathway (Supplementary Figure 1A, B). For the 15 negatively correlated DEGs of the gene sets, a total of 30 GO terms and 11 pathways (*P* < 0.05) were identified. The most significantly enriched biological process, cellular component, and molecular function of the GO terms were complement activation: the alternative pathway, platelet alpha granule lumen, and flavin adenine dinucleotide binding, respectively. Similarly, the significant pathways were mainly enriched in the tyrosine metabolism signalling pathway (Supplementary Figure 1C, D).

### Integration of the PPI network and Cytoscape analysis

**Figure 5:**
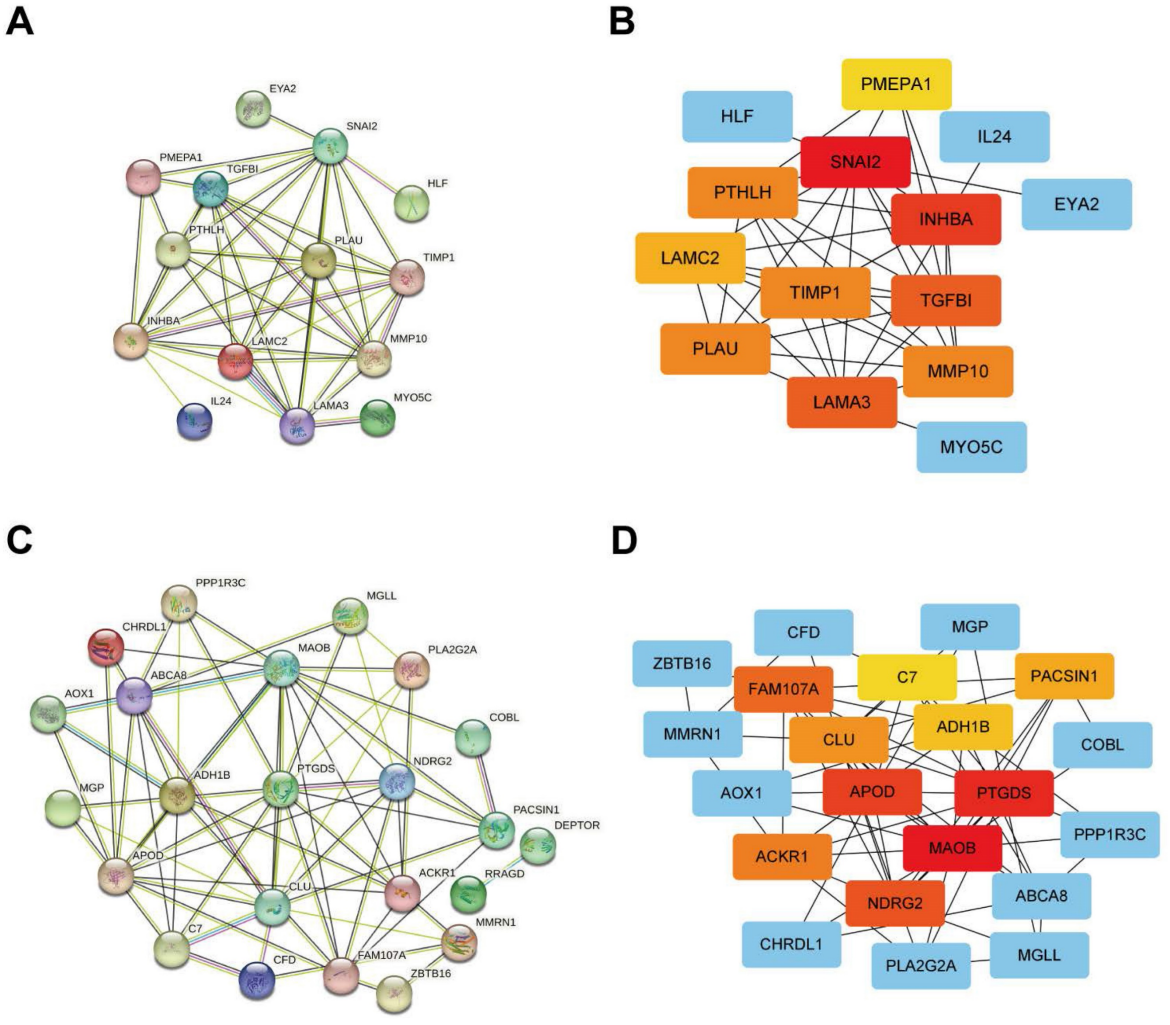
Protein-protein interaction network analysis of genes from different gene sets using the STRING online database and visualization using Cytoscape software. (A, B) PPI network analysis and Cytoscape analysis of gene sets that are positively associated with the tumour. (C, D) PPI network analysis and Cytoscape analysis of gene sets negatively associated with the tumour.

The outcomes of the PPI network of positively correlated DEG sets were obtained from STRING online analysis software, and Cytoscape software was used for visualization. The top 10 outstanding proteins (including proteins from similar families of hub genes on the Cytoscape website) were identified as hub genes and selected for further analysis (Figure 5A, B). In addition, the negatively correlated DEG sets were obtained from STRING online analysis software, and Cytoscape software was used for visualization. The top 10 outstanding proteins (including proteins from similar families of hub genes on the Cytoscape website) were identified as hub genes and selected for further analysis (Figure 5C, D).

### Expression levels and survival analysis of the hub genes by GEPIA

**Figure 6:**
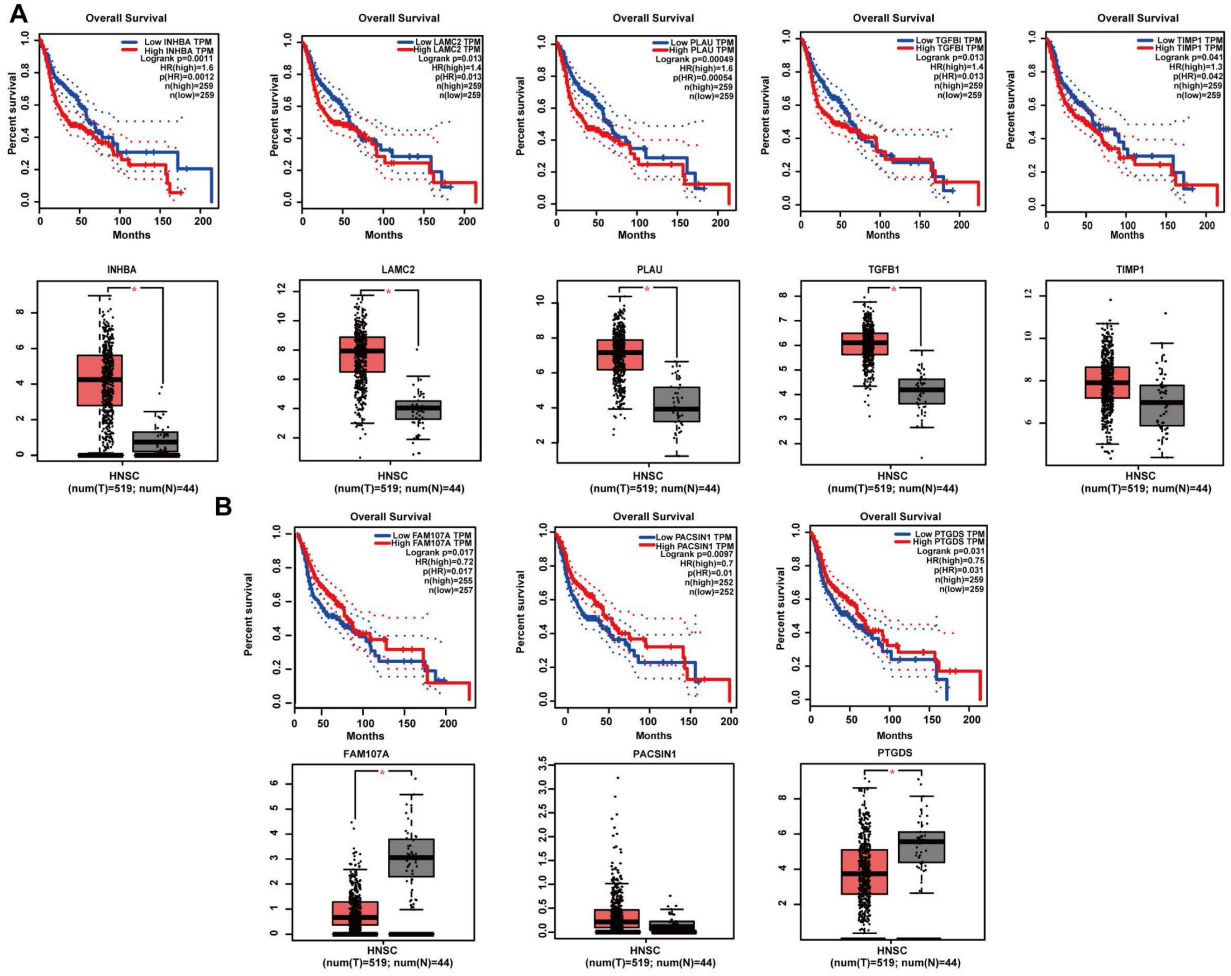
Survival analysis and expression levels of hub genes in different gene set analyses by the GEPIA online database. (A) INHBA, LAMC2, PLAU, TGFβ1, and TIMP1 were screened from the set of genes positively associated with tumours, and high expression of all five genes was associated with poor prognosis in HNSCC patients. The expression levels of INHBA, LAMC2, PLAU, and TGFβ1 were significantly higher in tumour tissues than in normal tissues, but there was no significant difference in the expression levels of TIMP1 between tumour tissues and normal tissues. (B) FAM107A, PACSIN1, and PTGDS were screened from the set of genes negatively associated with tumours, and high expression of all five genes was associated with better prognosis in HNSCC patients. The expression levels of FAM107A and PTGDS were significantly lower in tumour tissues than in normal tissues, but there was no significant difference in the expression levels of PACSIN1 between tumour tissues and normal tissues.

GEPIA online software was used to perform survival analysis of the 20 potential hub genes of 2 gene sets. The results revealed the significance of 8 potential hub genes as prognostic factors of patients with HNSCC. Specifically, for the positively correlated DEGs, high expression levels of INHBA (*P* = 0.0011), LAMC2 (*P* = 0.013), PLAU (*P* = 0.00049), TGFβ1 (*P* = 0.013) and TIMP1 (*P* = 0.042) were strongly associated with poor prognosis. In addition, except for TIMP1 (*P* > 0.05), the expression levels of INHBA (*P* < 0.05), LAMC2 (*P* < 0.05), PLAU (*P* < 0.05) and TGFβ1 (*P* < 0.05) were significantly different between tumour and normal samples (Figure 6A). For the negatively correlated DEGs, high expression levels of FAM107A (*P* = 0.017), PACSIN1 (*P* = 0.0097) and PTGDS (*P* = 0.031) were strongly associated with better prognosis. In addition, except for PACSIN1 (*P* > 0.05), the expression levels of FAM107A (*P* < 0.05) and PTGDS (*P* < 0.05) were significantly different between tumour and normal samples (Figure 6B).

### The expression levels of hub genes in HNSCC and normal samples

**Figure 7:**
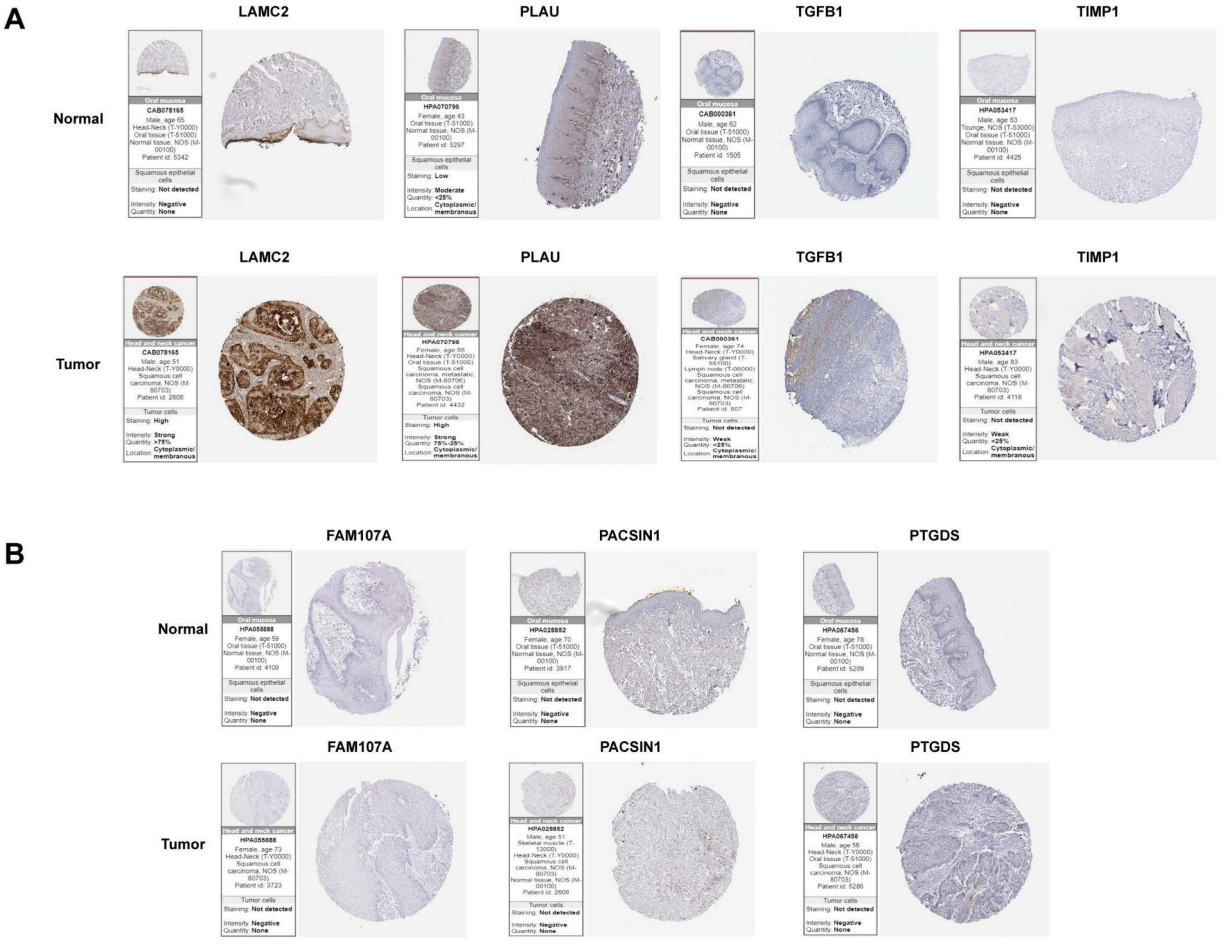
Differential expression levels of hub genes in tumour and normal tissues in two gene sets analysed by the HPA online database. (A) The expression levels of LAMC2, PLAU, TGFβ1 and TIMP1 in tumour and normal tissues. (B) The expression levels of FAM107A, PACSIN1 and PTGDS in tumour and normal tissues.

To further analyse the protein levels of the hub genes, the HPA database was used to determine the expression levels and locations of the hub genes. Since IHC results were not available for INHBA in the HPA online database, we excluded it. Among the positively correlated gene sets, PLAU and LAMC2 were more strongly expressed in tumour samples than in normal samples. TGFβ1 and TIMP1 expression was negative, and there was no significant difference between tumour and normal samples (Figure 7A). For the negatively correlated gene sets, FAM107A, PACSIN1, and PTGDS expression was negative, and there was no significant difference between tumour and normal samples (Figure 7B). This study mainly focuses on the screening and identification of genes related to a poor prognosis in patients with HNSCC. Based on the above results, PLAU and LAMC2 were selected for further studies.

### IHC staining of PLAU and LAMC2 in HNSCC samples

**Figure 8:**
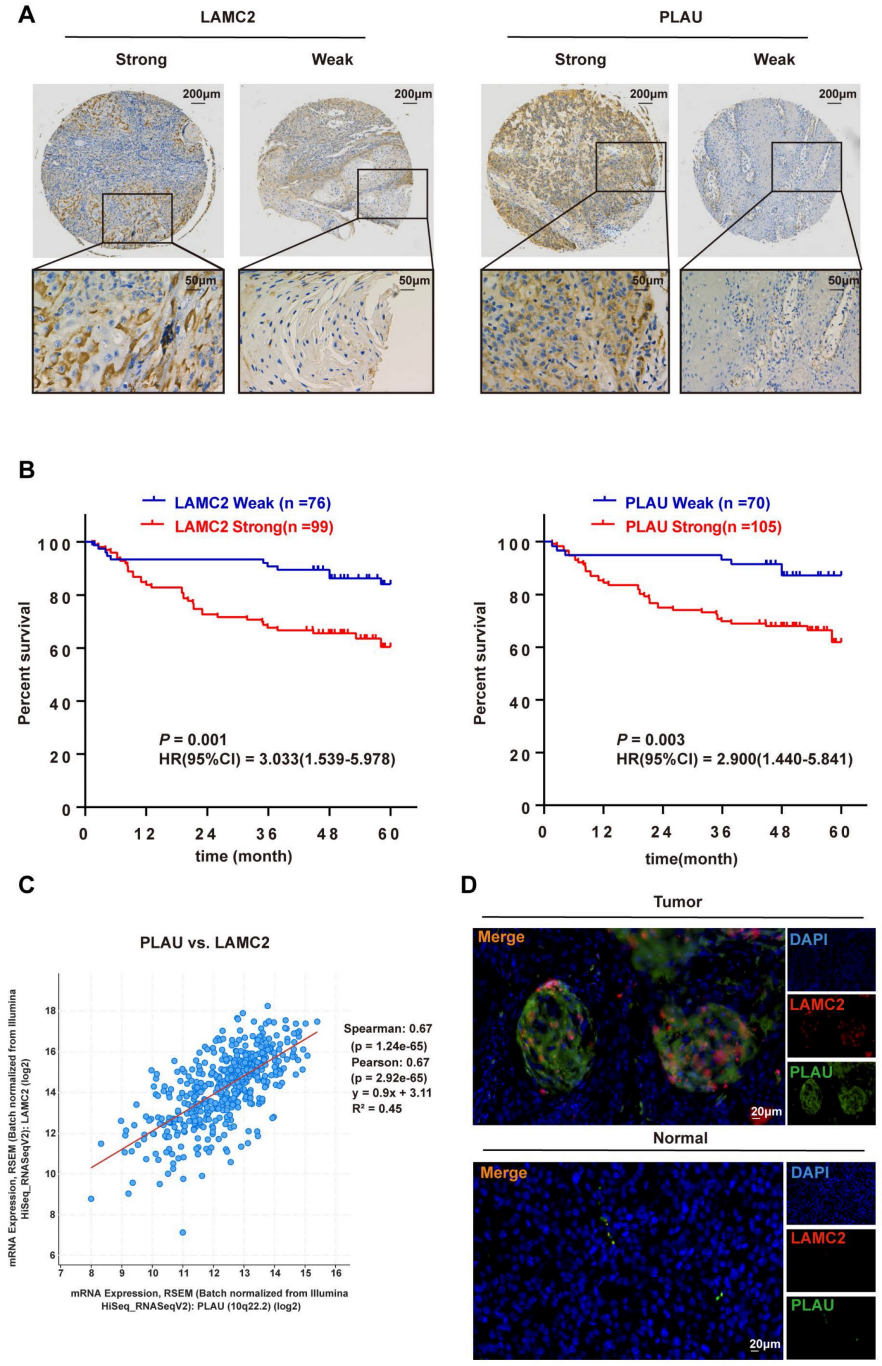
Correlation analysis of the differential expression levels of PLAU and LAMC2 with clinicopathological parameters in HNSCC patients. (A) The expression levels of PLAU and LAMC2 in 175 HNSCC patients. (B) Prognosis analysis between the differential expression levels of PLAU and LAMC2 in 175 patients with HNSCC. (C) Significant positive correlation between PLAU and LAMC2 expression in HNSCC samples according to the cBioPortal database. (D) Colocalization of PLAU and LAMC2 expression in HNSCC samples verified by immunofluorescence double-labelling.

The IHC results of 175 HNSCC samples showed that PLAU and LAMC2 were positively expressed, and there was a difference between strong and weak expression in HNSCC samples (Figure 8A).

### PLAU and LAMC2 independently predict the survival of 175 HNSCC patients

To further evaluate the impact of PLAU and LAMC2 in HNSCC patients, the association between the expression of PLAU and LAMC2 and patients' clinicopathological parameters was investigated. In total, 175 primary HNSCC patients were divided into two groups according to the expression levels of PLAU and LAMC2. High PLAU and LAMC2 expression was found to be correlated with clinical indicators (Table 1). Furthermore, multivariate Cox regression analysis showed that PLAU and LAMC2 were independent prognostic factors for HNSCC patients (Table S1 and Table S2 and Figure 8B).

### High expression of both PLAU and LAMC2 can a poor prognosis in HNSCC patients

A significant positive correlation between PLAU and LAMC2 expression in HNSCC samples was found based on the results of the TCGA database through gene correlation analysis via the cBioPortal database (Figure 8C). A positive correlation between PLAU and LAMC2 expression was also found in 175 HNSCC patients (contingency coefficient = 0.558, *P* < 0.001, Table S3). In addition, a clear colocalization expression relationship between PLAU and LAMC2 was found by immunofluorescence double-labelling in HNSCC samples (Figure 8D).

## Discussion

HNSCC is one of the most prevalent tumours, with approximately 550,000 new patients diagnosed yearly worldwide 21. This kind of tumour is characterized by high morbidity, a high risk of recurrence, regional cervical metastasis, and a poor prognosis 22. Currently, despite progress in treatment intervention and large numbers of basic studies, the prognosis and survival rate have not improved significantly 23. Increasing research has illustrated that the abnormal expression of genes is one of the risk factors for the occurrence and progression of HNSCC, and some dysregulated genes in HNSCC might be potential candidate biomarkers for prognosis 24. Therefore, our study aimed to investigate gene expression through bioinformatics analysis of the GSE23036 and TCGA datasets to identify significant molecules that might be used as biomarkers and therapeutic targets in HNSCC.

In the present study, a total of 952 DEGs were identified (293 upregulated and 659 downregulated) in the TCGA dataset, and a total of 570 DEGs were identified (236 upregulated and 334 downregulated) in the GEO dataset. WGCNA was performed for modular gene analysis of the DEGs in the 2 databases. In the TCGA and GEO datasets, 4 highly relevant expression patterns were identified, which are represented in the blue, red, green, and black modules. In the TCGA database, the blue module (positively correlated with tumour status) and the red module (negatively correlated with tumour status) were selected for further analysis.

In addition, in the GEO database, the green module (positively correlated with tumour status) and the black module (negatively correlated with tumour status) were selected for further analysis. Twenty-four genes positively correlated with tumour status and 15 genes negatively correlated with tumour status were subjected to GO and KEGG analyses. In the set of genes positively correlated with tumour status, GO term analysis showed that the hub genes were mainly enriched in the regulation of the extrinsic apoptotic signalling pathway in the absence of ligands, laminin complexes, and extracellular matrix structural constituents. KEGG pathway analysis revealed that the hub genes were mainly enriched in ECM-receptor interaction. Debodipta Das et al 25 found that the ECM-receptor pathway was significantly enriched in HNSCC patients, and the expression of some key genes (COL4A1, COL4A2, COL4A6, and LAMC2) in this pathway may be used as prognostic biomarkers for HNSCC. In the set of genes negatively correlated with tumour status, GO term analysis showed that the hub genes were mainly enriched in complement activation: the alternative pathway, platelet alpha granule lumen, and flavin adenine dinucleotide binding. KEGG pathway analysis revealed that the hub genes were mainly enriched in tyrosine metabolism. Brandon Leonard et al 26 showed that the tyrosine metabolism pathway was associated with chemotherapeutic resistance to cetuximab in HNSCC.

Moreover, according to the PPI network and Cytoscape analysis, we identified 20 hub genes in the 2 gene sets. Based on the prognosis analysis using the GEPIA online analysis tool, high expression levels of INHBA, LAMC2, PLAU, TGFβ1, and TIMP1 were strongly associated with poor prognosis in HNSCC patients. In addition, high expression levels of FAM104A, PACSIN1, and PTGDS were strongly associated with a better prognosis in HNSCC patients. Moreover, we used the HPA online database to determine the expression levels of 8 genes in HNSCC and normal samples. Finally, we found that the expression of PLAU and LAMC2 was consistent with the prognostic analysis results. LAMC2 is overexpressed in oesophageal squamous cell carcinoma (ESCC) samples and facilitates ESCC progression and metastasis, which is correlated with the poor prognosis of ESCC patients 27, however, we did not find any relevant high-quality research studies on LAMC2 in HNSCC. PLAU may be an independent prognostic biomarker for HNSCC, and downregulation of miR-23b-3p contributes to the tumorigenic effect of PLAU in HNSCC 28. In our study, PLAU and LAMC2 were first screened out by bioinformatics methods, and we found that PLAU and LAMC2 were highly expressed in HNSCC and were associated with poor prognosis. Therefore, we included the two genes in the subsequent analysis. IHC analyses of 175 HNSCC samples showed that PLAU and LAMC2 were positively expressed in HNSCC samples and that high expression levels of PLAU and LAMC2 were associated with age, T stage, N stage, clinical stage, and survival status. Moreover, univariate and multivariate Cox regression analyses demonstrated that high expression levels of PLAU and LAMC2 might be risk factors for poor prognosis in HNSCC, and the expression levels of PLAU and LAMC2 were positively correlated in HNSCC samples.

In conclusion, the present study successfully screened out PLAU and LAMC2, and comprehensive analysis showed that high expression of both PLAU and LAMC2 might predict a poor prognosis in patients with HNSCC.

## Supplementary Material

Supplementary tables and figure.

## Author contributions

JBT and ZCG led the team and were responsible for whole project administration. SLJ and SJN carried out all the experiments and wrote the manuscript. HC and YLX contributed to sample collection and validated the results. ZCG and SLJ analysed data and provided advice. All authors have read and approved the manuscript.

## Availability of data and materials

The datasets used and analysed during the current study are available from the corresponding author on reasonable request.

## Ethics approval and consent to participate

The study was conducted in accordance with the Declaration of Helsinki and approved by the Ethics Committee of College of Stomatology affiliated Xi'an Jiaotong University (approval no. xjkqll[2022]NO.028). Written informed consent was obtained from all patients for use of tissue sample. The TNM stage, clinicopathological classification and tumour stage of patients with HNSCC were assessed according to the American Joint Committee on Cancer (AJCC) guidelines.

## Figures and Tables

**Table 1:** Immunohistochemical expression of PLAU and LAMC2 in samples from 175 patients with HNSCC according to clinical data and follow‑up.

Variable		LAMC2		*P*	PLAU		*P*
		Weak	Strong		Weak	Strong	
Gender				0.951			0.694
	Male	51(43.6)	66(56.4)		48(41)	69(59)	
	Female	25(43.1)	33(56.9)		22(37.9)	36(62.1)	
Age				0.001^**^			0.000 ^***^
	<60 years	35(60.3)	23(39.7)		34(58.6)	24(41.4)	
	≥60 years	41(35)	76(65)		36(30.8)	81(69.2)	
Differentiation				0.206			0.409
	Poor- Moderately	25(51)	24(49)		22(44.9)	27(55.1)	
	Well	51(40.5)	75(59.5)		48(38.1)	78(61.9)	
T Stage				0.001^**^			0.001^**^
	T1-2	58(53.2)	51(46.8)		54(49.5)	55(50.5)	
	T3-4	18(27.3)	48(72.7)		16(24.2)	50(75.8)	
N Stage				0.005^**^			0.033^*^
	N0	32(59.3)	22(40.7)		28(51.9)	26(48.1)	
	N+	44(36.4)	77(63.6)		42(34.7)	79(65.3)	
Clinical Stage				0.002^**^			0.025^*^
	I-II	27(64.3)	15(35.7)		23(54.8)	19(45.2)	
	III-IV	49(36.8)	84(63.2)		47(35.3)	86(64.7)	
Recurrence				0.065			0.663
	No	49(49.5)	50(50.5)		41(41.4)	58(58.6)	
	Yes	27(35.5)	49(64.5)		29(38.2)	47(61.8)	
Living Status				0.001^**^			0.002^**^
	Living	65(50.8)	63(49.2)		60(46.9)	68(53.1)	
	Dead	11(23.4)	36(76.6)		10(21.3)	37(78.7)	

*P < 0.05, **P < 0.01, ***P < 0.001.
