# Supplementary material for: PLAU and LAMC2 can predict a poor prognosis in patients with HNSCC
Source: J Cancer. 2023 Jun 4;14(9):1660–72. doi: 10.7150/jca.84407 (PMC10266254; doi:10.7150/jca.84407)
Supplement: Supplementary file 1 — Supplementary tables and figure. [file jcav14p1660s1.pdf]

Table S1. Univariate COX regression analysis of influencing factors of HNSCC patients death

| Variable | | β | SE | Wald$\chi^2$ | HR(CI 95%) | *P* |
|---|---|---|---|---|---|---|
| Gender | Male | | | | 1.000 | |
| | Female | -0.025 | 0.308 | 0.006 | 0.976(0.533-1.784) | 0.936 |
| Age | <60 years | | | | 1.000 | |
| | ≥60 years | 0.723 | 0.357 | 4.099 | 2.06(1.023-4.147) | 0.043* |
| Differentiation | Poor- Moderately | | | | 1.000 | |
| | Well | -0.704 | 0.295 | 5.685 | 0.495(0.277-0.882) | 0.017* |
| T Stage | T1-2 | | | | 1.000 | |
| | T3-4 | 0.603 | 0.293 | 4.239 | 1.827(1.029-3.244) | 0.039* |
| N Stage | N0 | | | | 1.000 | |
| | N+ | 1.088 | 0.41 | 7.035 | 2.968(1.328-6.63) | 0.008** |
| Clinical Stage | I-II | | | | 1.000 | |
| | III-IV | 0.891 | 0.438 | 4.143 | 2.437(1.034-5.744) | 0.042* |
| Recurrence | No | | | | 1.000 | |
| | Yes | 1.177 | 0.313 | 14.114 | 3.244(1.756-5.994) | <0.001*** |

*$P < 0.05$, **$P < 0.01$, ***$P < 0.001$.

Table S2. Correlation analysis of the expression levels of LAMC2 and PLAU in HNSCC patients deaths.

| Variable | | Model1 | | Model2 | |
|---|---|---|---|---|---|
| | | HR (95% CI) | *P* | HR (95% CI) | *P* |
| LAMC2 | Weak | 1.000 | | 1.000 | |
| | Strong | 3.033(1.539-5.978) | 0.001** | 2.304(1.050-5.059) | 0.037* |
| PLAU | Weak | 1.000 | | 1.000 | |

| | | | | |
|---|---|---|---|---|
| Strong | 2.900(1.440-5.841) | 0.003** | 2.389(1.115-5.118) | 0.025* |

No indicators were adjusted in Model 1, indicators such as age, T stage, and recurrence were adjusted in Model 2, **P*< 0.05, ***P*< 0.01.

Table S3. Correlation analysis of the expression levels of the relationship between LAMC2 and PLAU in 175 HNSCC patients

| LAMC2 | PLAU | | *P* |
|---|---|---|---|
| | Weak | Strong | |
| Weak | 59(77.6) | 17(22.4) | <0.001*** |
| Strong | 11(11.1) | 88(88.9) | |

***$P$< 0.001.

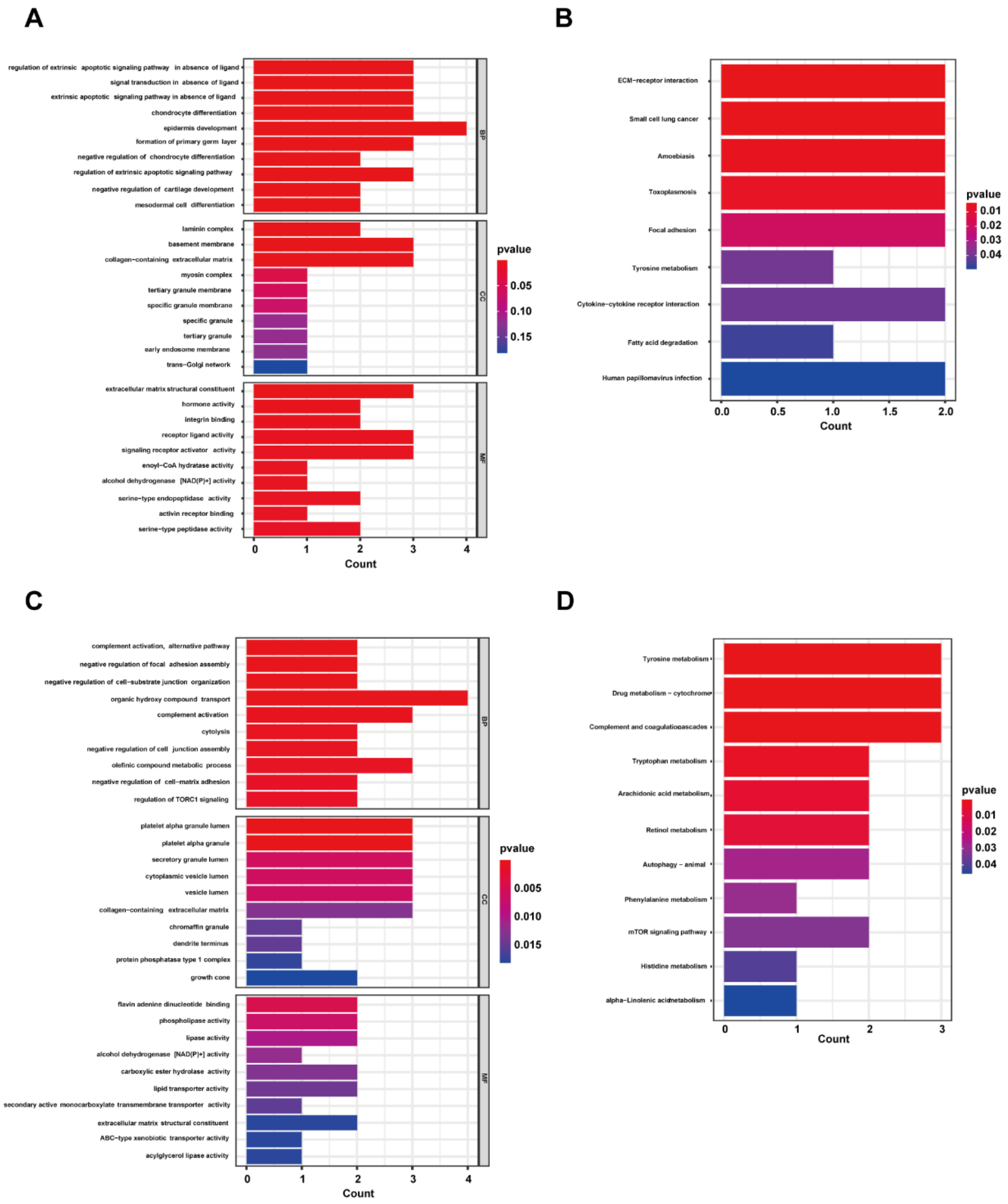


Supplementary Figure 1: Functional enrichment analysis of different gene sets by GO and KEGG. (A, B) GO and KEGG enrichment analyses of gene sets that are positively associated with the tumour. (C, D) GO and KEGG enrichment analyses of gene sets that are negatively associated with the tumour.
